# Association of immunonutrition-based indices (Hemoglobin-Albumin-Lymphocyte-Platelet, prognostic nutritional index, and C-reactive protein–albumin–lymphocyte) with the latency period in preterm prelabor rupture of membranes

**DOI:** 10.1590/1806-9282.20251977

**Published:** 2026-06-29

**Authors:** Ilayda Gercik Arzik, Hakan Golbasi, Hale Ankara Aktas, Ilknur Toka, Zubeyde Emiralioglu Cakir, Ceren Saglam Purut, Raziye Torun, Emre Bayram, Burak Bayraktar, Atalay Ekin

**Affiliations:** 1 Izmir City Hospital, Department of Perinatology – Izmir, Turkey.; 2 University of Health Sciences, Ankara Etlik City Hospital, Department of Perinatology – Ankara, Turkey.; 3 University of Health Sciences, Ankara Etlik City Hospital, Department of Obstetrics and Gynecology, Division of Perinatology – Ankara, Turkey.

**Keywords:** Fetal membranes, Premature rupture, Nutrition assessment, Inflammation, Infant, premature, Pregnancy outcome

## Abstract

**OBJECTIVE:**

The aim of this study was to evaluate the predictive value of immunonutrition-based indices (Hemoglobin, Albumin, Lymphocyte, Platelet score, prognostic nutritional index, and C-reactive protein–albumin–lymphocyte index) for latency duration and neonatal outcomes in preterm prelabor rupture of membranes.

**METHODS:**

This retrospective cohort study included 145 singleton pregnancies diagnosed with PPROM between 24 and 34 weeks. Patients were categorized by latency duration (≤48 vs. >48 h), and subgroup analyses used thresholds of 72 h, 96 h, and 7 days. Maternal, laboratory, and neonatal characteristics were compared. Logistic regression adjusted for parity and gestational age evaluated associations between indices and latency.

**RESULTS:**

Hemoglobin, Albumin, Lymphocyte, Platelet, prognostic nutritional index, and C-reactive protein–albumin–lymphocyte values showed no significant differences across latency groups (all p>0.05). Only respiratory distress syndrome was more frequent in the short-latency group (p=0.005).

**CONCLUSION:**

Immunonutrition indices remained stable across latency durations, highlighting the predominance of localized inflammatory and structural mechanisms in preterm prelabor rupture of membranes pathophysiology.

## INTRODUCTION

Preterm prelabor rupture of membranes (PPROM) accounts for about one-third of all preterm births and remains a major contributor to neonatal morbidity and mortality. It complicates approximately 2–3% of all pregnancies^1^. It is defined as the spontaneous rupture of fetal membranes before 37 weeks of gestation and prior to the onset of labor^2^. The latency period, representing the interval between membrane rupture and delivery, plays a critical role in determining perinatal outcomes. While prolongation of this period allows time for antenatal corticosteroid, antibiotic, and neuroprotective therapy, it also increases the risk of chorioamnionitis and neonatal sepsis^3^. The timing of corticosteroid and neuroprotective therapy in PPROM cases also affects neonatal outcomes; however, there is no marker for predicting the time of delivery in expected management cases^4^.

The pathophysiology of PPROM is multifactorial, involving the disruption of membrane integrity through mechanical, biochemical, and inflammatory processes. Inflammatory mediators such as cytokines, proteases, and oxidative stress molecules play key roles in membrane weakening^5^. Several serum and local biomarkers, including interleukin-6 (IL-6), C-reactive protein (CRP), matrix metalloproteinases (MMPs), and cell-free DNA (cfDNA), have been investigated for predicting latency and infection risk; however, their clinical use remains limited due to inter-laboratory variability, invasiveness, and inconsistent accuracy^6-8^. This highlights the need for simple and noninvasive parameters applicable in daily practice.

Recent studies have shown that several immunonutrition-based indices may have prognostic importance in obstetric and gynecologic conditions^9,10^. These indices are calculated from routine blood tests and reflect the body's overall inflammatory, immune, and nutritional status. Among them, the hemoglobin–albumin–lymphocyte–platelet (HALP) score, the prognostic nutritional ındex (PNI), and the C-reactive protein–albumin–lymphocyte (CALLY) index are increasingly recognized as practical markers that combine these biological factors into a single measure. They have been studied in disorders such as preeclampsia, hyperemesis gravidarum, and gynecologic cancers, but their potential role in PPROM has not yet been clarified^11,12^.

In this study, we aimed to examine the association and predictive efficacy of immunonutrition-based indices (HALP, PNI, and CALLY) with the duration of the latency period and neonatal outcomes in PPROM cases.

## METHODS

### Study design and population

This retrospective cohort study was conducted at the Department of Perinatology, University of Health Sciences, Izmir City Hospital, Izmir, Turkey, between November 2023 and August 2025. A total of 145 singleton pregnant women diagnosed with PPROM between 24 and 34 weeks of gestation were included, as delivery is generally recommended after 34 weeks of gestation in accordance with current clinical guidelines^13^. All antenatal follow-ups, deliveries, and neonatal care were performed at the same institution.

### Case and control selection

Patients were divided into two groups according to the latency period, defined as the interval (in hours) between membrane rupture and delivery: ≤48 h (n=70) and >48 h (n=75). Additional subgroup analyses were performed using thresholds of 72 h, 96 h, and 7 days. The latency period was calculated as the time interval between the reported onset of membrane rupture, based on patient history and clinical assessment, and delivery.

Patients with a clinical history suggestive of membrane rupture and/or ultrasonographic evidence of oligohydramnios were evaluated by sterile speculum examination. PPROM was diagnosed **only when objective confirmation was obtained**, defined as visible pooling of amniotic fluid in the posterior vaginal fornix and/or a positive immunochemical swab test based on insulin-like growth factor binding protein-1 (IGFBP-1). This test detects high concentrations of IGFBP-1 in amniotic fluid and has been reported to have a sensitivity of 86–87.5% and a specificity of 74–94.4% for the diagnosis of membrane rupture. Cases without objective confirmation were not considered as PPROM and were excluded from the study^14,15^.

Patients with fetal anomalies, multiple gestations, chronic maternal diseases, or regular medication use were excluded. Those with major obstetric complications such as preeclampsia, HELLP syndrome, placenta previa, or placental abruption, as well as medically indicated preterm deliveries or incomplete clinical or laboratory data, were also excluded from the analysis.

To ensure that the duration of the latency period was evaluated independently of infection-related early deliveries, patients showing clinical signs suggestive of intraamniotic infection (IAI) were excluded. Accordingly, women presenting with maternal fever (≥38°C), uterine tenderness, or fetal tachycardia at admission were not included in the study.

### Clinical management and treatment protocols

All patients diagnosed with PPROM received standard antibiotic therapy within the first 6 h of hospital admission. The treatment protocol consisted of 1 g oral azithromycin and 2 g intravenous ampicillin (every 6 h for 2 days), followed by 875 mg oral amoxicillin twice daily for 5 days.

In accordance with institutional protocol, all patients received antenatal corticosteroid therapy for fetal lung maturation (two intramuscular doses of 12 mg betamethasone 24 h apart). Oral nifedipine was administered for short-term tocolysis only in patients with uterine activity suggestive of threatened preterm labor, with the aim of completing the antenatal corticosteroid course (a 30 mg loading dose followed by 10 mg every 6 h for 48 h). In addition, in pregnancies between 24+0 and 31+6 weeks of gestation, magnesium sulfate was administered for fetal neuroprotection when preterm delivery was anticipated, in accordance with international guidelines^16^.

### Data collection and laboratory evaluation

All clinical, laboratory, and obstetric data were retrospectively collected from the hospital electronic medical records. Recorded parameters included maternal age, gravidity, parity, body mass index (BMI), gestational age at admission and delivery, mode of delivery, and neonatal outcomes.

Laboratory parameters included hemoglobin, lymphocyte, neutrophil, platelet, albumin, and CRP levels. All measurements were obtained from blood samples drawn at the time of hospital admission, prior to the initiation of any medication.

The following immunonutrition-based indices were calculated using the respective formulas:

- HALP=Hemoglobin (g/dL) × Albumin (g/L) × Lymphocyte (10^9^/L)/Platelet (10^9^/L)
- PNI=Albumin (g/L)+5×Lymphocyte (10^9^/L)
  CALLY=Albumin (g/L)×Lymphocyte (10^9^/L)/CRP (mg/dL)

### Statistical analysis

A power analysis was performed using G*Power 3.1 software to evaluate the statistical power of the study. The effect size was set at a moderate level (d=0.5), the significance level at 5% (α=0.05), and the test power at 95% (1-β=0.95). Based on these parameters, the sample size was determined to provide sufficient statistical power.

All statistical analyses were performed using IBM SPSS Statistics version 26 (IBM Corp., Armonk, NY, USA). The normality of continuous variables was assessed using the Kolmogorov-Smirnov and Shapiro-Wilk tests. Normally distributed data were expressed as mean±standard deviation (SD) and compared using the independent samples t-test, whereas non-normally distributed data were presented as median (interquartile range, IQR) and compared using the Mann-Whitney U test. Categorical variables were presented as numbers and percentages (n, %) and compared using the chi-square or Fisher's exact test, where appropriate.

To further investigate the relationship between immunonutrition-based indices and latency intervals (≤48 vs. >48 h, ≤72 vs. >72 h, ≤96 vs. >96 h, and ≤7 vs. >7 days), binary logistic regression analyses were performed. Latency duration was entered as the dependent variable, and each index (HALP, PNI, and CALLY) was evaluated as an independent variable. Regression models were adjusted for parity and gestational age at hospitalization as potential confounders. Adjusted analyses were performed using analysis of covariance (ANCOVA), with parity and gestational age at hospitalization included as covariates. Results were presented as crude and adjusted p-values, and a two-tailed p-value of <0.05 was considered statistically significant.

### Ethical considerations

Our study was approved by the İzmir City Hospital Non-Interventional Clinical Research Ethics Committee (Approval No: 2025/244, Date: May 21, 2025). The study was conducted in accordance with the ethical principles outlined in the Declaration of Helsinki. Due to the retrospective design of the study and the use of anonymized patient data, the requirement for informed consent was waived by the ethics committee. No external financial support was received for this study, and the authors declare that they have no conflicts of interest related to the conduct or publication of this work.

## RESULTS

A total of 145 women with PPROM diagnosed before 34 weeks of gestation were included in the analysis. The study population was divided into two groups based on latency duration: ≤48 h (n=70, 48.3%) and >48 h (n=75, 51.7%).

The clinical characteristics of the patients and their distribution within the groups are listed in Table 1. There were no significant differences between the two groups regarding maternal age, BMI, nulliparity rate, or conception by assisted reproductive techniques. However, gravidity and parity were significantly higher in the prolonged latency group. Gestational age at hospitalization was higher in the short latency group (p<0.001), while gestational age at delivery did not differ significantly. Birth weight was significantly higher in the prolonged latency group (p=0.010). The majority of deliveries in both groups were performed by cesarean section, without a significant difference (p=0.318).

**Table 1:** Maternal characteristics, perinatal and neonatal outcomes of the participants.

	Latency period ≤48 h n=70 (48.3%)	Latency period >48 h n=75 (51.7%)	p-value
Maternal age (years)	30.3±6.5	30.5±6.7	0.894
Gravidity	1 (1)	2 (3)	0.010
Parity	0 (1)	1 (2)	0.049
Nulliparous	40 (57.1%)	34 (45.3%)	0.155
In vitro fertilization	1 (1.4%)	2 (2.6%)	0.616
BMI (kg/m^2^)	27.7±4.7	28.3±4.8	0.403
Gestational age at hospitalization (weeks)	30.2±3.5	27.4±3.2	<0.001
Gestational age at delivery (weeks)	31±3.3	32±2.7	0.102
Mode of delivery			0.318
Vaginal birth	24 (34.3%)	20 (26.6%)	
Cesarean section	46 (65.3%)	55 (73.7%)	
Birth weight (gram)	1,643±642	1,977±614	0.010
Apgar score at 1st min	7 (3)	7 (2)	0.120
Apgar score at 5th min	8 (1)	8 (1)	0.340
Transient tachypnea of the newborn	7 (10%)	13 (17.3%)	0.200
Respiratory distress syndrome	53 (75.7%)	40 (53.3%)	0.005
Continuous positive airway pressure	35 (50%)	33 (44%)	0.469
Mechanical ventilation	16 (22.8%)	15 (20%)	0.675
Neonatal sepsis	8 (11.4%)	9 (12.8%)	0.914
Neonatal hyperbilirubinemia	25 (35.7%)	28 (37.3%)	0.839
Phototherapy for neonates	23 (32.8%)	27 (36%)	0.690
NICU admission	66 (94.3%)	69 (92%)	0.587
Perinatal mortality	3 (4.3%)	3 (5.3%)	0.931

BMI: body mass index; NICU: neonatal intensive care unit. Data are expressed as mean±SD, median (interquartile range), or n, % where appropriate.

Neonatal outcomes were largely similar between the groups. **The 1- and 5-min Apgar scores** did not differ between groups, and the rates of transient tachypnea, neonatal sepsis, hyperbilirubinemia, and phototherapy requirement were similar across groups. However, respiratory distress syndrome was observed more frequently in the short latency group (p=0.005). Admission to the neonatal intensive care unit (NICU) and perinatal mortality rates were high in both groups but did not differ significantly (both p>0.05).

Laboratory parameters at the time of membrane rupture are summarized in Table 2. Levels of hemoglobin, white blood cells, neutrophils, lymphocytes, platelets, albumin, and CRP were similar in both groups, with no significant differences.

**Table 2:** Comparison of laboratory parameters between preterm prelabor rupture of membranes patients with latency periods ≤48 and >48 h.

	Latency period ≤48 h n=70 (48.3%)	Latency period >48 h n=75 (51.7%)	p-value
Hemoglobin (g/dL)	11.3±0.8	11.1±0.8	0.125
White blood cell count (10^9^/L)	12.26±3.19	12.19±4.43	0.934
Neutrophil count (10^9^/L)	8.96 (3.95)	8.73 (4.04)	0.484
Lymphocyte count (10^9^/L)	1.96±0.56	2±0.65	0.730
Platelet count (10^9^/L)	239±70	247±66	0.572
Albumin (g/L)	36.8±2.7	36.2±3.7	0.652
CRP (mg/dL)	7 (16)	6 (10.5)	0.148

CRP: C-reactive protein. Data are expressed as mean±SD or median (interquartile range) where appropriate.

The immunonutrition-associated indices (HALP, PNI, and CALLY) were evaluated across multiple latency thresholds (≤48 h, ≤72 h, ≤96 h, and ≤7 days). In all subgroup analyses, mean and median values were similar, and no statistically significant differences were detected (all p>0.05, Table 3). After adjustment for parity and gestational age at hospitalization, the results remained unchanged, indicating that these variables did not confound the associations.

**Table 3:** Comparative analysis of immunonutrition-associated inflammatory indices among preterm prelabor rupture of membranes patients stratified by latency intervals.

	Latency period ≤ 48 h n=70 (48.3%)	Latency period >48 h n=75 (51.7%)	p-value	Adjusted p-value*	Latency period ≤ 72 h n=84 (57.9%)	Latency period >72 h n=61 (42.1%)	p-value	Adjusted p-value*
CALLY	10.64 (17.58)	12.04 (19.34)	0.222	0.435	11.1 (17.58)	10.62 (19.1)	0.958	0.994
HALP	3.61±1.19	3.41±1.33	0.447	0.176	3.45±1.17	3.67±1.49	0.441	0.689
PNI	36.88±2.74	36.23±3.74	0.334	0.891	36.54±3.22	36.54±3.53	0.999	0.283
	**Latency period ≤96 h n=94 (64.8%)**	**Latency period >96 h n=51 (35.2%)**	**p-value**	**Adjusted p-value** *	**Latency period ≤7 days n=110 (75.9%)**	**Latency period >7 days n=35 (24.1%)**	**p-value**	**Adjusted p-value** *
CALLY	11 (19.21)	10.32 (18.9)	0.942	0.995	10.99 (17.95)	11.92 (21.29)	0.408	0.413
HALP	3.51±1.32	3.51±1.1	0.989	0.776	3.54±1.30	3.25±0.92	0.499	0.079
PNI	36.43±3.24	36.88±3.51	0.564	0.817	36.42±3.19	37.6±4.19	0.284	0.904

CALLY: CRP-albumin-lymphocyte index; HALP: Hemoglobin, Albumin, Lymphocyte, Platelet score, PNI: prognostic nutritional index. Data are expressed as mean±SD or median (interquartile range) where appropriate.

* p-values were adjusted for parity and gestational age at hospitalization.

Overall, the immunonutrition-based indices demonstrated stable values across different latency durations in PPROM, indicating consistent biochemical profiles regardless of latency length.

## DISCUSSION

PPROM remains one of the most significant problems in obstetric practice and represents a leading cause of neonatal morbidity and mortality. The duration of the latency period has a direct impact on neonatal outcomes and is therefore a critical factor in clinical management. Prolongation of this period offers opportunities for antenatal corticosteroid administration, antibiotic prophylaxis, and preparation for delivery. Consequently, ­reliable biomarkers are needed to predict this period. In this study, we investigated the potential role of immunonutrition-related indices in predicting the latency period among patients with PPROM. In this study, immunonutrition-based indices (HALP, PNI, and CALLY) were not associated with latency duration across multiple clinically relevant cut-off points in pregnancies complicated by PPROM, and neonatal outcomes were largely comparable between latency groups.

Immunonutrition-based indices (HALP, PNI, and CALLY) are noninvasive, easily calculable parameters that reflect both the systemic inflammatory response and nutritional status. As they can be derived from routine laboratory tests, their use in obstetric practice has recently gained attention. In the literature, HALP and PNI have been primarily evaluated for predicting the severity of preeclampsia, the risk of preterm labor, and the presence or severity of hyperemesis gravidarum, where they have been shown to reflect the systemic inflammatory response^9,11,17^. Additionally, these indices have been reported as independent prognostic markers for survival in gynecologic malignancies^18^. The CALLY index, a newer composite parameter incorporating inflammatory and immune components, has so far been evaluated only in the context of gynecologic cancers^12^. Considering the inflammation-based pathophysiology of PPROM, it has been predicted that these systemic indices may have potential value in predicting the duration of the latency period. However, to the best of our knowledge, no previous studies have examined these indices specifically in PPROM, making our study the first of its kind in the literature.

Several studies and reviews have highlighted that PPROM and spontaneous preterm labor (sPTL) represent distinct clinical entities with differing underlying mechanisms, particularly in terms of inflammation and immune response^19,20^. Nevertheless, many studies evaluating latency duration have grouped PPROM and sPTL together under the broader term "spontaneous preterm birth" (sPTB)^21,22^. This lack of distinction between subtypes complicates the identification of specific predictive patterns unique to each condition. In line with this distinction, elevated levels of cfDNA have been associated with inflammation-related sPTB, reflecting increased cellular turnover and immune activation. However, in PPROM, cfDNA appears to be more closely related to localized membrane inflammation and structural damage rather than a generalized systemic inflammatory burden, which may partly explain the limited predictive value of systemic immunonutritional indices for latency duration^8,23^.

Among hematological and inflammatory indices investigated for latency prediction in sPTB, the neutrophil-to-lymphocyte ratio (NLR) and platelet-to-lymphocyte ratio (PLR) have been most frequently reported^24,25^. These parameters have been associated with shorter latency and have predicted delivery within 7 days with moderate to high accuracy (area under the curve [AUC] ranging from 0.68 to 0.98)^11,25^. Gezer et al. reported that NLR>6.2 significantly predicted delivery within seven days (AUC=0.71) in a cohort of more than 400 cases^26^. Hrubaru et al. also demonstrated predictive value for NLR and PLR (AUC=0.694 and 0.682, respectively) in a cohort of 243 preterm cases^21^. However, in both studies, PPROM cases were not analyzed separately and represented only a small subset of participants.

Similarly, the CXCL10/CCL2 ratio has shown high predictive accuracy for delivery before 34 weeks of gestation (AUC=0.83; sensitivity 67%, specificity 86%)^27^. Other systemic inflammatory markers, such as the CRP/albumin ratio (CAR) and fibrinogen/albumin ratio (FAR) have also been investigated for latency prediction; however, in most studies, it was either unclear whether PPROM cases were included or they were analyzed together with sPTL^28,29^. Therefore, large-scale, prospective studies that separately evaluate PPROM and sPTL are warranted to clarify these differences.

When focusing solely on PPROM, most available studies have aimed to predict chorioamnionitis, neonatal sepsis, or histologic infection rather than latency duration^30,31^. These studies can be categorized according to the biological sample used: maternal serum, cervicovaginal fluid, or amniotic fluid. Maternal serum–based studies have reported associations between latency duration and biomarkers such as CRP, IL-6, endotoxin activity, and oxidative stress markers, suggesting that they may predict delivery within 3–7 days^28,32^. Cervicovaginal or vaginal fluid studies have mainly focused on MMP-8, beta-human chorionic gonadotropin (β-hCG), alpha-fetoprotein (AFP), prolactin, urea, and creatinine levels, which have been proposed as potential markers of shorter latency^33,34^. Amniotic fluid–based studies have identified interleukin-8 (IL-8), MMP-9, lipocalin-2, and S100A8/A9 as mediators associated with early delivery^35^. Nevertheless, none of these biomarkers have demonstrated sufficient accuracy or consistency for routine clinical application.

This discrepancy in biomarker studies can largely be explained by the different pathophysiological basis of sPTL and PPROM. While sPTL is primarily driven by systemic inflammation, reduced progesterone action, uteroplacental ischemia, or uterine overdistension, PPROM results from the structural weakening of fetal membranes^36^. MMP-mediated collagen degradation, localized inflammation, and subclinical infection are considered the main contributors to this process^37,38^. Therefore, while indices reflecting the systemic inflammatory response may be significant in preterm labor, their predictive power may be limited in PPROM. In our study, immunonutrition-based indices (HALP, PNI, and CALLY) were evaluated across different latency thresholds (≤48 h, ≤72 h, ≤96 h, and ≤7 days), and none demonstrated statistically significant differences between groups (all p>0.05). Our findings confirm that local inflammatory processes and membrane integrity disruption, rather than systemic inflammation, are the determining factors in the pathophysiology of PPROM.

Several points should be considered when interpreting our results. First, monitoring all patients at the same center and under similar antibiotic and corticosteroid protocols may have reduced variability in systemic inflammatory responses and masked potential differences. Moreover, HALP, PNI, and CALLY primarily reflect chronic nutritional and immune status and may be insufficient to capture the acute, localized inflammatory response associated with membrane rupture. Future prospective studies incorporating dynamic biochemical assessments are needed to address these limitations.

Despite these constraints, our study has several notable strengths, including a homogeneous patient population, standardized management protocols, and stratified analyses across multiple latency intervals. Nonetheless, our study is the first to evaluate HALP, PNI, and CALLY indices specifically in the context of PPROM, representing an important contribution to the literature.

## CONCLUSION

Our findings did not show statistically significant associations, yet they offer clinically meaningful insight. Systemic immunonutrition indices seem to have limited value in predicting latency duration in PPROM. This stability across varying latency periods suggests that localized inflammatory and structural mechanisms, rather than systemic changes, play a dominant role in its pathophysiology. Future research should aim to identify biomarkers that better reflect these local processes to improve predictive accuracy and clinical applicability.

## ETHICS APPROVAL STATEMENT

This study complied with all relevant national regulations, institutional policies, and the principles of the Helsinki Declaration (as revised in 2013). The study protocol was reviewed and approved by the İzmir City Hospital Ethics Committee (Approval number: 2025/244).

## Data Availability

The datasets generated and/or analyzed during the current study are available from the corresponding author upon reasonable request.
